# Virus-like Particle Vaccines of Infectious Bursal Disease Virus Expressed in *Escherichia coli* Are Highly Immunogenic and Protect against Virulent Strain

**DOI:** 10.3390/v15112178

**Published:** 2023-10-30

**Authors:** Pengchao Ji, Tiantian Li, Yanan Wu, Qi Zhao, Lu Li, Xuejian Shi, Wenting Jiang, Jiabin Wang, Panpan Wang, Tingting Wang, Dawei Jiang

**Affiliations:** 1 College of Veterinary Medicine, Henan Agricultural University, Zhengzhou 450046, China; pengchaoji@henau.edu.cn (P.J.); tiantianli0113@126.com (T.L.); wlyananjiayou@yeah.net (Y.W.); 18838980392@163.com (Q.Z.); li18338215039@163.com (L.L.); m18703610387@163.com (X.S.); jiang18860354181@163.com (W.J.); wangjiabin0923@163.com (J.W.); wangpan11191001@163.com (P.W.); w202215670775495@163.com (T.W.); 2 International Joint Research Center of National Animal Immunology, College of Veterinary Medicine, Henan Agricultural University, Zhengzhou 450046, China; 3 Longhu Laboratory, Zhengzhou 450046, China

**Keywords:** infectious bursal disease virus (IBDV), vaccine, adjuvant, immune, virus-like particles (VLPs)

## Abstract

Objectives: Infectious bursal disease virus (IBDV) is a highly contagious, acutely infectious agent that causes immunosuppression in chickens. We expressed IBDV VP2 proteins in *Escherichia coli* (*E. coli*) to develop an effective virus-like-particles (VLPs) vaccine and evaluated its immunogenicity. Methods: The VLPs produced in *E. coli* were used as an immunogen mixed with a water-in-mineral-oil adjuvant (Montanide^TM^ ISA 71 VG, ISA 71 RVG) or a white oil (7#) adjuvant. VLPs without an adjuvant, commercial subunit vaccine, inactivated vaccine, and attenuated vaccine were used as controls. These test vaccines were intramuscularly injected into 19-day-old SPF chickens, which were challenged with the IBDV virulent strain at 30 days after vaccination. Results: The adjuvants boosted antibody production, and the adjuvant groups (except white oil) produced higher antibody levels than the non-adjuvanted controls and the commercial vaccine groups. In terms of cellular immunity, the VLPs plus adjuvant combinations produced higher levels of cytokines, IL-2, IL-4, and IFN-γ than the controls. Conclusion: IBDV VLPs plus the ISA 71 RVG adjuvant can be used as an optimal vaccine combination for improving the immune efficacy of IBD subunit vaccines, which can protect against the virulent strain.

## 1. Introduction

IBDV is responsible for the acute, contagious, and immunosuppressive infectious bursal disease (IBD) in chicks that causes huge economic losses in the chicken industry worldwide [1]. IBDV mainly infects the central immune organ bursa of chicks and destroys immature B lymphocytes, ultimately causing immunosuppression, susceptibility to other microorganisms, and vaccination failure [2]. The viral genome consists of two segments of double-stranded RNA, A and B, which encode five viral proteins, VP1, VP2, VP3, VP4, and VP5 [3]. The VP2 protein is a major host-protective antigen containing epitopes responsible for inducing neutralizing antibodies against IBDV [4]. The VP2 protein is the major structural component of the IBDV icosahedral capsid [5] and can spontaneously form particles that enhance immune protection [6,7,8].

At present, VP2 for use in developing subunit vaccines has been produced in different expression systems, such as *Escherichia coli* [9], *baculovirus* [10], *yeast* [11], and *plants* [12]. Although the *baculovirus* system is the most frequently used to produce IBDV VLPs, it is inefficient, low-yield, and high cost [10,13]. In comparison, *E. coli* expression systems are more convenient, economical, and fast for the production of recombinant proteins on an industrial scale. Studies have shown that recombinant VP2 proteins can be efficiently expressed in *E. coli* and self-assembled into VLPs [13]. The IBDV VP2 subunit vaccine has been widely tested [8,9,10,11,12,13,14,15], but its immunogenicity was limited because *E. coli* lacks the ability to produce modifications after protein expression. VLPs are a powerful tool for enhancing the immunogenicity of IBDV subunit vaccines because they are similar in structure to natural viral capsids and are effective at inducing a humoral response [16]. Therefore, production of IBDV VP2 VLPs using the *E. coli* expression system affords a potential strategy for the development of IBDV subunit vaccines.

Research has shown that when VP2 is mixed with various adjuvants, such as oil [12], Freund’s adjuvant [13], or Taishan Pinus massoniana pollen polysaccharide (TPPPS) [17], it can produce higher antibody levels and better protection. At present, the Montanide^TM^ ISA series of water-in-oil emulsion adjuvants, which has been widely used in animal models, is very effective at enhancing the humoral and/or cellular immune response and producing a stronger protective effect than traditional adjuvants [18]. Previous studies have shown that ISA 71 VG adjuvants are safe and effective mainly through enhancing the cellular immune response with the production of cytokines, IFN-γ, IL-2, and IL-4 [19]. Using the optimal adjuvant improves the immunogenicity of VLPs.

In this study, we developed an economical and effective subunit vaccine against IBDV using the VP2 protein expressed and self-assembled into VLPs in an *E. coli* expression system. The VLPs were then mixed with different adjuvants and used as an immunogen. The test vaccines were intramuscularly injected into 19-day-old SPF chickens, after which they were challenged with the standard virulent strain BC6/85 of IBDV and their protective effects were evaluated. Comparison allowed us to optimize the vaccine/adjuvant combination, which offers better options for IBD subunit vaccines.

## 2. Materials and Methods

### 2.1. Expression and Purification of the VP2 Protein

Recombinant *Escherichia coli* containing the VP2 gene fragment of the IBDV B87 strain (Henan Key Laboratory of Animal Immunology, China, GenBank ID: DQ202329.1) were grown in 150 mL of fresh LB medium in the presence of kanamycin (100 μg/mL) in a shaker (200× *g*) to an OD600 of 0.5 at 37 °C, then induced with 0.2 mM of isopropyl-β-D-thiogalactopyranoside (IPTG) for 21 h at 37 °C [20]. Cells were harvested using centrifugation at 8000× *g* for 10 min at 4 °C, resuspended in phosphate-buffered saline (PBS, pH 7.4), and then disrupted using sonication. The cell lysates containing VP2 proteins were centrifuged at 10,000× *g* for 20 min at 4 °C and supernatants were collected. Proteins were precipitated with the addition of saturated ammonium sulfate to the supernatants, dissolved in Tris buffer (20 mM Tris-HCl, pH 8.0), and purified using anionic ion-exchange chromatography (IEC) and hydrophobic interaction chromatography (HIC) (GE HealthCare, Freiburg, Germany) according to the manufacturer’s instructions. The purity and bioactivity of the proteins were determined with 12% SDS-PAGE and Western blot, respectively. Western blot analysis was performed with anti-His tag mouse monoclonal antibody (Qiagen, Shanghai, China) and goat anti-mouse IgG antibody (Abbkine, Atlanta, GA, USA).

### 2.2. VLP Assembly and Quantification

The purified VP2 proteins in PBS were assembled into VLPs. Nickel grids (200-mesh) with a carbon-coated collodion film were placed upside down on a drop of VLP suspension for 2 min, after which the grids were washed with PBS and stained with 3% (*w*/*v*) phosphotungstic acid (pH 6.8). The VP2 protein assemblies were observed under a transmission electron microscope (JEM-1400, Hitachi Ltd., Tokyo, Japan) and imaged to indicate shape and size. The size and uniformity of the VLPs in suspension were measured with dynamic light scattering (DLS).

### 2.3. Vaccine Preparation

Purified protein solutions were filtered through a 0.22 μm membrane and quantified with a BCA protein assay kit (Invitrogen, Beijing, China). Aliquots of VLPs (0.05 mg/mL) were mixed with Montanide^TM^ ISA 71 VG and ISA 71 RVG (Seppic, Paris, France) at a 30:70 ratio (w:w) and with white oil adjuvant at 50:50, and emulsified by vortexing for 5 min. The commercial vaccines were used according to the manufacturer’s instructions.

### 2.4. Animal Immunization and Serum Sampling

All animal experiments were approved by the Animal Ethics Committee of the Animal Experiment Committee of the Henan Academy of Agricultural Sciences (Approval number LLSC41022052). To mitigate the potential impact of the immunogen on the physical and mental well-being of birds during the experiment, a total of 135 specific-pathogen-free (SPF) white Leghorn chickens (19 days old) were procured from Spencer (Shandong, China) and housed in negative-pressure filtered-air isolators. They were sacrificed using the cervical dislocation method under xanthine anesthesia to reduce the pain of the birds.

The immunization program is shown in Table 1. A total of 135 19-day-old SPF chickens were randomly divided into 9 groups of 15 birds each. Group A was immunized intramuscularly with 200 μL VLPs (10 μg) without adjuvant. Groups B, C, and D were immunized intramuscularly with 200 μL VLPs (10 μg) with ISA 71 VG, ISA 71 RVG, and white oil (7#) adjuvants, respectively. As controls, groups E and F were immunized intramuscularly with 300 μL of commercial IBDV subunit vaccine (Qingdao Yebio Biological Engineering Co. Ltd., Qingdao, China) and inactivated IBDV vaccine (Huahong Biology, China), respectively. Group G was immunized orally with 300 μL of attenuated IBDV (B87 strain) (Guangxi Liyuan Biological Co. Ltd., Nanning, China). Group H was immunized intramuscularly with 200 μL of PBS, and neither vaccinated nor challenged (as negative control). Blood samples were collected from the wing vein at 0, 7, 14, 21, and 28 days after vaccination, and the sera were collected at 1500× *g*.

### 2.5. Viral Challenge Study

The IBDV standard virulent strain BC6/85 (China Institute of Veterinary Drug Control, Beijing, China) was used for the challenge. On the fourth day after the challenge, we strangled the chicken to suffocate and remove it quickly. Under visual observation, the bursa of Fabricius appeared swollen or showed clear yellow exudation, jelly samples, or bleeding. We then determined the chicken’s infectious bursal disease by viewing typical lesions. The challenge virus was prepared by orally inoculating SPF chickens at five weeks of age and harvesting the bursae at three days post-infection. The bursae were homogenized in PBS (1:10 ratio) and stored at −80 °C.

The statistical analysis of the BC6/85 strain BID_50_ was conducted following Karber’s method of biological statistics, as described in *Veterinary Microbiology* by the Harbin Veterinary Research Institute, Chinese Academy of Agricultural Sciences (China Agriculture Press, December 1998, pp. 596–597). Calculation formula: logBID_50_ = L − d (S − 0.5). In the formula: L = logarithm of the lowest dilution, d = the difference between the logarithms of dilution, and S = total rate of bursal lesions.

All groups except group I (negative control) were challenged at 30 days after vaccination via the oral route with 10^−5^ BID_50_ of the standard virulent IBDV strain BC6/85, and the chickens were observed for clinical symptoms and survival status over 4 days. At 4 days post-infection, the chickens were euthanized and necropsied, and the leg muscles, bursae of Fabricius, and other major organs (liver and kidney) were examined for pathogenic gross lesions. The bursae to body weight (BF/BW) ratio was calculated as bursae of Fabricius weight/body weight ×1000. 

### 2.6. Determination of Serum Antibody Titers

Sera were obtained using low-speed centrifugation of clotted blood samples. The VP2-specific antibody titer was determined using a commercial antibody ELISA (CK113) test kit (BioChek, Denbighshire, UK), following the manufacturer’s instructions. The absorbance was determined with a microplate reader at a wavelength of 405 nm. 

### 2.7. Detection of Cytokine Production

The IFN-γ, IL-2, and IL-4 levels in the serum before challenging the chickens were determined with ELISA using IFN-γ (SEA049Ga), IL-4 (SEA077Ga), and IL-2 (SEA073Ga) kits (Cloud-Clone Corp., Katy, TX, USA), following the manufacturer’s instructions. The absorbance at 450 nm was determined with a microplate reader. 

### 2.8. Histopathology Examination 

The bursae of Fabricius were removed and fixed in formalin for scoring of the histopathological lesion severity (Table 2). The efficacy of protection was determined by calculating the ratio of chickens exhibiting histopathological BF lesion scores ranging from 1 to 4 to the total number of chickens within each group.

### 2.9. Statistical Analysis

Analysis of variance (ANOVA) and other statistical analyses (antibody titers and cytokines) were performed using GraphPad Prism version 5.00 (GraphPad Software, San Diego, CA, USA). Data are shown as the mean ± SD, and statistical significance was set at *p* < 0.05 for all tests.

## 3. Results

### 3.1. Expression and Purification of IBDV VP2 Protein

**Figure 1:**
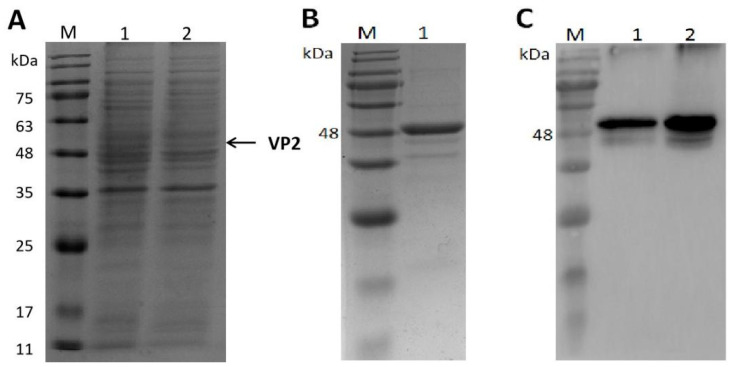
Expression and purification of IBDV VP2 protein. (**A**) SDS-PAGE analysis of the expression of VP2 protein. Lane M, protein marker; Lane 1, cell lysate after induction; Lane 2, cell containing vector mock after induction. (**B**) SDS-PAGE analysis of the purified VP2 protein after ion-exchange chromatography and hydrophobic interaction chromatography. Lane M, protein marker; Lane 1, purified VP2 protein. (**C**) Western blot analysis of VP2 protein with anti-His monoclonal antibody. Lane M, protein marker; Lane 1, cell lysate after induction; Lane 2, the purified VP2 protein.

The expression of the IBDV VP2 protein was confirmed using SDS-PAGE and Western blot (Figure 1A,C). The SDS-PAGE results showed that VP2 protein with a molecular weight of approximately 50 kDa was produced under induction with IPTG, and the immunoblot results confirmed that the target band in SDS-PAGE was VP2. After purification with IEC and HIC, the purity of the VP2 protein was estimated to be > 90% (Figure 1B,C); the concentration of purified VP2 protein was 3.5 mg/L as determined using BCA protein assay; and the yield of VP2 from the *E. coli* culture was 0.054 mg/L.

### 3.2. Assembly and Characterization of VP2 VLPs

**Figure 2:**
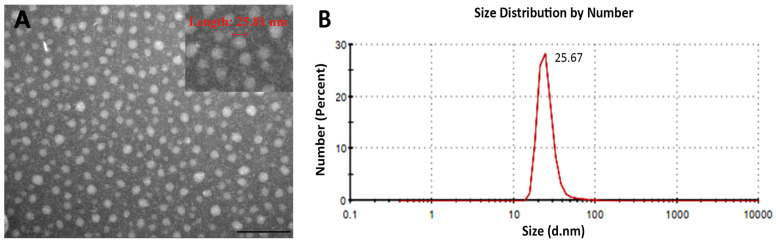
Assembly and characterization of VLPs from purified VP2 protein. (**A**) Confirmation of VLPs using transmission electron microscopy (TEM) in PBS. Bar represents 200 nm; the upper right part of the picture is a bit zoomed to see 25 nm VLPs. (**B**) Dynamic light scattering (DLS) results of the VLPs assembled.

Previous studies have shown that VP2 proteins can self-assemble into VLPs with a diameter of 25 nm [21]. To determine whether the VP2 proteins expressed by *E. coli* were capable of forming VLPs after purification with IEC and HIC, the purified VP2 proteins were analyzed using TEM and DLS. TEM images of the purified VP2 revealed that it did self-assemble into VLPs (Figure 2A). The DLS results showed that the VLP diameter was about 25 nm and the size distribution was highly uniform (Figure 2B).

### 3.3. The Humoral Immunity of IBDV VLPs

**Figure 3:**
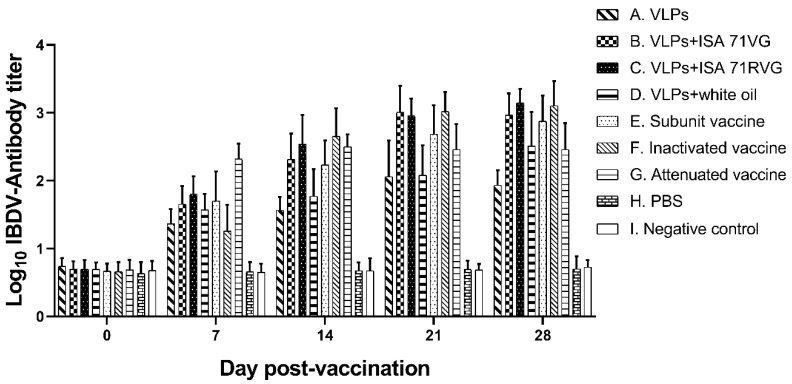
IBDV-specific immune response in chickens. Nineteen-day-old SPF chickens in nine groups were vaccinated with A VLPs, B VLPs plus ISA 71 VG, C VLPs plus ISA 71 RVG, D VLPs plus white oil adjuvant, E commercial subunit vaccine, F inactivated vaccine, G attenuated vaccine, H PBS, and I non-vaccinated. The sera were collected at 0, 7, 14, 21, and 28 dpv. The ELISA antibody titers for each group are represented as the mean ± SD on a log10 scale.

The antibody titers against IBDV were determined with ELISA at 0, 7, 14, 21, and 28 days post-vaccination (dpv). The mean serum antibody titers in each group are shown in Figure 3. From 7 to 28 dpv, all vaccinated groups had significantly higher antibody titers than the control (PBS) and negative groups (*p* < 0.01). The groups receiving VLPs plus adjuvant (B, C, and D) produced antibodies earlier than those given VLPs alone or inactivated vaccine, and they had higher antibody titers than the VLP-only group. The antibody titers in the VLPs with adjuvant groups continued to increase from 0 to 28 dpv, but the antibody levels in the VLP group only showed a downward trend on day 28 compared with day 21. On day 28 pv, the antibody titers in the groups receiving VLPs plus the three adjuvants were: ISA 71 RVG (log10^3.14^) > ISA 71 VG (log10^2.96^) > white oil (log10^2.51^). Furthermore, the antibody titers recorded for the commercial vaccine group were as follows: inactivated vaccine (log10^3.10^) > subunit vaccine (log10^2.87^) > attenuated vaccine (log10^2.46^). In addition, the antibody titers of the ISA 71 RVG group were similar to those of the inactivated vaccine group and were higher than those of the commercial subunit vaccine group and the attenuated vaccine group. The ISA 71 RVG group had higher antibody titers than the groups given commercial vaccines (subunit vaccine, inactivated vaccine, and attenuated vaccine). The results demonstrate that the *E. coli*-synthesized VLPs had good immunogenicity, that ISA adjuvants seem to be more efficient than white oil, and that it is as efficient as commercial vaccines, which are really good results.

### 3.4. The Cellular Immunity of IBDV VLPs

**Figure 4:**
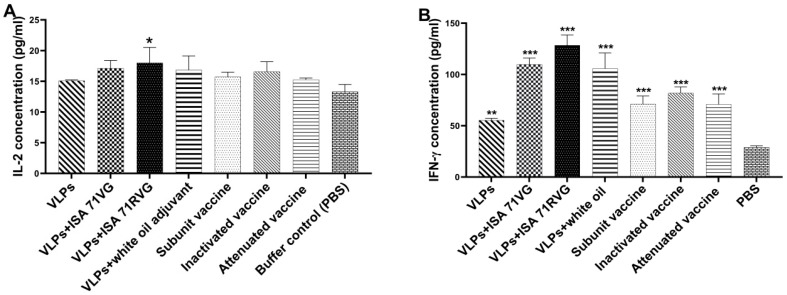
Analysis of cytokines in immunized chickens. Serum was collected at 28 dpv, and (**A**) IL-2 and (**B**) IFN-γ concentrations were determined with ELISA kits. All values shown are means ± SD; statistical differences between antigen groups and PBS group were measured with one-way ANOVA (*** *p* < 0.001, ** *p* < 0.01, * *p* < 0.05).

The IL-2, IL-4, and IFN-γ cytokine levels in serum samples from immunized chickens were evaluated with ELISA (Figure 4), and the statistical differences between the antigen groups and the PBS group were measured with one-way ANOVA (*** *p* < 0.001, ** *p* < 0.01, * *p* < 0.05). The results showed that there was no significant difference in IL-4 levels between the antigen groups and the PBS control. The IL-2 level in the VLP plus ISA 71 RVG group was significantly higher than that of the PBS group (* *p* < 0.05) only, but the difference was not significant with the other antigen groups (*p* > 0.05). For the IFN-γ levels, the antigen groups were significantly higher than the PBS group (** *p* < 0.01), and the VLP plus adjuvant groups were significantly higher than the commercial groups (subunit vaccine, inactivated vaccine, and attenuated vaccine) (* *p* < 0.05). In addition, the VLP-immunized groups exhibited the same trend in cytokine production, IFN-γ > IL-2 > IL-4, and the levels of all three cytokines in the VLP plus ISA 71 RVG group were a bit higher than in the other groups.

### 3.5. Protective Effect of VLPs against the Virulent IBDV Strain

**Figure 5:**
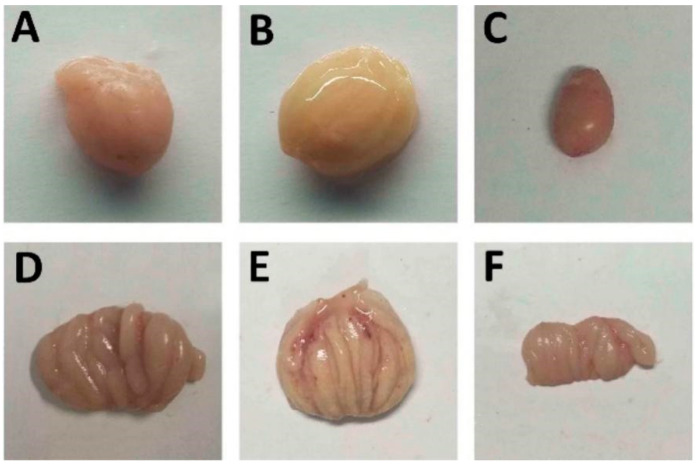
Gross morphology of bursae of chickens challenged with IBDV strain BC6/85. (**A**,**D**) Normal bursae from unvaccinated and unchallenged chickens. (**B**,**E**) Edematous, yellow jelly-like and hemorrhagic bursae from challenged chickens. (**C**,**F**) Atrophic bursae from attenuated vaccine group.

To test the protective efficacy of the vaccine, 49-day-old chickens were challenged with the standard virulent IBDV strain BC6/85 (10^−5^ BID_50_ per bird), except for group I. No mortality was observed in any group of chickens in this experiment. The chickens were dissected at four days post-challenge. Necropsy of the diseased chickens showed varying degrees of hemorrhage in the thigh muscles, bursal atrophy, cyst formation, yellow gelatinous coatings, or hemorrhages (Figure 5). The number of chickens in the VLP-only group with bursal lesions was significantly higher than that in the group receiving VLPs plus adjuvants (B, C, and D).

The BF/BW ratios, bursal lesion scores, and protection rates were also determined (Table 3). The VLP plus adjuvant groups (B, C, and D) showed significantly greater protection than the group with VLPs alone (*p* < 0.05), and the immune protection rate of the three adjuvants was: ISA 71 RVG (13/15) > ISA 71 VG (11/15) > white oil (10/15). The VLPs plus ISA 71 RVG produced a higher protection rate than the commercial subunit vaccine (10/15) and the attenuated vaccine (8/15). These results indicated that the combination of VLPs with ISA 71 RVG gave the best protection against IBDV. 

**Figure 6:**
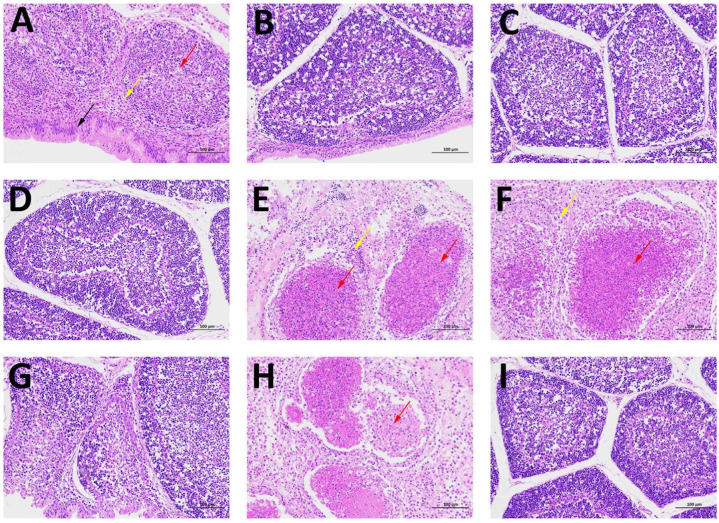
Histopathology of bursae of Fabricius of chickens from different groups stained with H&E (×200). Bursa follicles from chickens vaccinated with (**A**) VLPs, (**B**) VLPs plus ISA 71 VG, (**C**) VLPs plus ISA 71 RVG, (**D**) VLPs plus white oil, (**E**) subunit vaccine, (**F**) inactivated vaccine, and (**G**) attenuated vaccine; and (**H**) unvaccinated, after the virulent IBDV strain BC6/85 challenge. (**I**) Normal bursa follicle from unvaccinated and unchallenged birds. The brown arrow represents an increase in bursal epithelial cells and a small amount of heterophilic granulocyte infiltration. The red arrows represent the necrosis of the follicular cells, and a large number of disintegrating cell fragments are visible in the center of the follicular medulla. The yellow arrows indicate the disappearance of part of the follicular structure, inflammatory cell infiltration, and inflammatory exudate.

The pathological changes in the bursae of all groups were revealed with hematoxylin and eosin (H&E) staining (Figure 6). The bursal lesion scores based on bursal histopathological characteristics are presented in Table 3. There were no pathological symptoms observed in the bursae of groups B (VLPs plus ISA 71 VG), C (VLPs plus ISA 71 RVG), D (VLPs plus white oil), or I (unchallenged). The bursal lesions of the group receiving VLPs without adjuvant were mild, such as increased epithelia, decreased lymphocytes in the follicles, and inflammatory cell infiltration in the interstitial spaces. Bursal necrosis in the groups given inactivated or attenuated vaccine was severe: follicular cells were basically necrotic and some follicular structures had disappeared.

## 4. Discussion

The VP2 protein of IBDV is a major structural and host-protective protein and has been used as a target for the development of subunit vaccines. Compared with other expression systems, the *E. coli* expression system is more convenient, more economical, and faster for manufacturing owing to the ease of growth and genetic manipulation. Chelating Ni^2+^ Sepharose columns [22], sucrose-gradient centrifugation [23], and cesium chloride gradient centrifugation [24] are often used to obtain higher-purity VP2 proteins, but these purification processes are not suitable for large-scale production. In this study, VP2 protein was successfully expressed in *E. coli* cells and purified with saturated ammonium sulfate precipitation, IEC, and HIC; this guarantees the vaccine’s safety and ease of biological process amplification. The VP2 protein was purified to a level of 3.5 mg/L, which is significantly higher than previously reported [9]. The purified VP2 protein could form VLPs with a diameter of 25 nm (Figure 2), which was the same as that expressed in insect cells [11] and yeast cells [10]. Our results proved that the *E. coli* expression system and the purification method could produce high yields of IBDV VLPs.

Humoral immunity is the first layer of protection of the immune system against microbial attack. VLPs are effective in inducing humoral immune responses without adjuvant and can be used to improve the immunogenicity of antigens, and the addition of a chemical adjuvant may further promote the immune response. In this study, the animals receiving VLPs with adjuvants produced earlier and higher levels of antibodies than those receiving VLPs without adjuvants. Among the different adjuvants, ISA 71 RVG (antibody titer about log10^3.14^) produced the highest level of antibodies and had levels comparable to the three commercial vaccine groups (Figure 3). In addition, the dose (10 μg) and frequency (once) of immunization with VLPs were lower than those of the previous studies. These results indicate that combining VLPs with oil-based adjuvants not only improved the immune response but also reduced the dose of antigen and the frequency of immunization required. In addition, because the level of antibodies produced by the traditional attenuated vaccine was lower than that of the inactivated vaccine, the immune dose of the attenuated vaccine group increased in this experiment (1.5 feathers/1 mL). The experimental results showed that while the antibody level of the attenuated vaccine group increased, the increase caused bursal damage, which further confirmed its limited safety.

The secreted Th1 cytokines, IFN-γ and IL-2, induce T-cell growth and cytotoxic activity and mainly promote cell-mediated immune responses, while the Th2 cytokine, IL-4, mediates humoral immune responses [25]. In addition, IFN-γ can inhibit Th2 cell function and prevent the production of IL-4. In this experiment, the VLPs group had higher levels of IFN-γ and IL-2 than the PBS group, which confirms that IBDV VLPs stimulate cellular immune responses. The VLPs plus adjuvant groups had higher levels of IFN-γ and IL-2 than the VLPs-only group and the commercial vaccine (subunit, inactivated, and attenuated) groups (Figure 4B), which shows that the combination of VLPs and adjuvants stimulated the body to produce stronger cellular immunity. The levels of IFN-γ and IL-2 in the ISA 71 RVG adjuvant group were slightly higher than in the ISA 71 VG and white oil adjuvant groups, which shows that the ISA 71 RVG adjuvant produced the strongest Th1 response. This was consistent with the IBDV-induced immune response.

In this study, immunized chickens were challenged with the classic virulent IBDV BC6/85 strain, which causes immunosuppression but little or no mortality; the characteristic lesion is associated with bursitis leading to further bursal atrophy. In the challenge control group, we observed significant lesions (yellow gelatinous coating, indicative of bursal atrophy) in the bursae of Fabricius, and > 70% of the follicles were atrophied and necrotic. Follicular atrophy has been shown to cause immunosuppression. In the results of challenge protection, the adjuvant groups were significantly higher than the adjuvant-free group (Table 3). Both ISA 71 RVG and 71 VG showed good immune effects, but ISA 71 RVG could more effectively stimulate INF-γ and IL-2 to further enhance immune protection and afford better protection than the commercial vaccines. The study illustrates how a combination of adjuvant and VLPs could produce a higher level of protection than VLPs alone. The immune protection rate of each group was consistent with the increase and decline of antibody titer in each group, and cell-mediated immunity involving T cells appears to contribute to improving the protection against IBDV. In this experiment, the immune response and protection rate of the group receiving VLPs with no adjuvant were low, which may be due to the low dose of immunization.

## 5. Conclusions

In conclusion, this report documents the efficacy of different adjuvants combined with VLPs for IBDV subunit vaccination. In this experiment, *Escherichia coli* was used to express IBDV VP2 proteins, which could be isolated using convenient and low-cost purification methods and demonstrated the ability to self-assemble into VLPs that possessed immunogenicity. The results of animal experiments showed that the VLPs assembled by prokaryotic expression had good immunogenicity in combination with different adjuvants (Montanide^TM^ ISA 71 VG, ISA 71 RVG, white oil), and ISA 71 RVG was the optimum adjuvant for the IBDV VLPs. The VLPs, in combination with the ISA 71 RVG adjuvant, induced high levels of immune response and enhanced protection against IBDV. Our alternative vaccine against IBDV offers a new option for improving the immune efficacy of IBD subunit vaccines.

## Figures and Tables

**Table 1:** Experimental design for chicken immunization.

Group	Composition	Immunizing Dose	Administration	Number of Chickens	Age (Days) at Vaccination
A	10 μg VLPs	200 μL	Intramuscular	15	19
B	10 μg VLPs + ISA 71 VG	200 μL	Intramuscular	15	19
C	10 μg VLPs + ISA 71 RVG	200 μL	Intramuscular	15	19
D	10 μg VP2 + white oil (7#) adjuvant	200 μL	Intramuscular	15	19
E	Subunit vaccine ^a^ (AGP ≥ 16)	300 μL	Intramuscular	15	19
F	Inactivated vaccine	300 μL	Intramuscular	15	19
G	Attenuated vaccine (B87 strain)	1.5 feathers/1 mL	Oral	15	19
H	Challenged control (PBS)	200 μL	Intramuscular	15	19
I	-	-	-	15	-

^a^ The agar diffusion titer of the subunit vaccine is ≥1:16.

**Table 2:** Criteria of the histopathologic bursal lesion score.

Bursal lesion score	1	2	3	4
Follicular atrophy	0–10%	10–30%	30–70%	>70%

**Table 3:** Protection efficacy against virulent IBDV strain BC6/85.

Group	Vaccine	No. of Animals with BF Lesions ^a^	BF/BW Ratio ^b^	No. of Animals with BF/BW Ratio <2 ^c^	Histopathological BF Lesion Score ^d^				Protection ^e^
					1	2	3	4	
A	VLPs	11/15	3.80 ± 1.62	0/15	3	3	6	3	4/15
B	VLPs + ISA 71 VG	4/15	3.73 ± 0.64	0/15	11	2	1	1	11/15
C	VLPs + ISA 71 RVG	2/15	3.70 ± 0.82	0/15	13	1	1	0	13/15
D	VLPs + white oil	5/15	3.32 ± 0.44	0/15	10	3	1	1	10/15
E	Subunit vaccine	5/15	3.88 ± 0.66	0/15	10	3	1	1	10/15
F	Inactivated vaccine	2/15	3.49 ± 0.57	1/15	12	2	1	0	13/15
G	Attenuated vaccine	7/15	2.32 ± 0.57	4/15	1	3	1	2	8/15
H	Challenged control	15/15	3.42 ± 0.77	0/15	0	0	0	15	0/15
I	Unchallenged control	0/15	3.62 ± 0.73	0/15	15	0	0	0	15/15

^a^ Number of birds exhibiting severe BF lesion/number of birds. ^b^ Average of BF/BW ratio (bursa of Fabricius weight/body weight) × 1000 ± SD of the birds. ^c^ Number of animals with BF/BW ratio lower than 2/number of birds. BF/BW ratio lower than 2 indicates bursal atrophy. ^d^ The bursa lesion score was calculated according to a previously described method. ^e^ Protection assessment is based on bursal lesions score of 1 and BF/BW ratio < 2. Expressed as the number of protected chickens/total number of chickens in a group.

## Data Availability

All available data are presented in this manuscript.
